# Nasogastric Tube-Induced Catastrophic Airway Compromise Due to a Large Blood Clot

**DOI:** 10.7759/cureus.34472

**Published:** 2023-01-31

**Authors:** Elliot Runge, Salman Mohammed, Mutsumi Kioka

**Affiliations:** 1 Pulmonology and Critical Care, University of Nevada Las Vegas School of Medicine, Las Vegas, USA; 2 Critical Care Medicine, University of Nevada Las Vegas School of Medicine, Las Vegas, USA; 3 Pulmonary and Critical Care Medicine, University of Nevada Las Vegas School of Medicine, Las Vegas, USA

**Keywords:** cognitive defect, nose bleed, difficult airway management, nasogastric tube complications, aspiration hazard

## Abstract

Nasogastric and orogastric tube (NGT/OGT) insertion is a routine in-hospital procedure used in patients who need enteral feeding, medication administration, and gastric decompression in a patient unable to tolerate per oral administration. NGT insertion has a relatively low complication rate when performed adequately; however, previous studies demonstrate that associated complications range from delicate, simple nose bleeds to more severe conditions such as nasal mucosal bleeding, which can be easily aspirated in a patient with encephalopathy or other conditions associated with the inability to protect the airway. Here we present a case of traumatic NGT insertion causing nasal bleeding, leading to respiratory distress secondary to aspiration of blood clot obscuring the airway.

## Introduction

While routinely inserted without complication, nasogastric tube (NGT) and orogastric tube (OGT) mispositioning are well-documented in the literature with a spectrum of intra-thoracic and extra-thoracic complications [1-3]. These prior highlighted case reports focused on pulmonary complications such as pneumothorax and tracheobronchial perforation; however, many other complications can arise. Other injuries can occur, ranging from simple nose bleeds to life-threatening epistaxis [4].

Nasogastric tubes can cause mucosal injury, with subsequent nasal bleeding secondary to that injury, and then possible aspiration of blood in a patient unable to protect their airway. This is especially true in a patient with underlying coagulopathy, as described in this case. Other insults have been reported, such as rupture of the cribriform plate, esophageal perforation, and even traumatic intracranial insertion in the setting of previous craniofacial surgery [5].

Multiple guidelines/recommendations have been published in the literature to mitigate these risks and to distinguish between the correct and incorrect placement of NGT [6]. However, none of these recommendations account for the traumatic nasal mucosal injury described in this case, highlighting the need for physician awareness of this possible complication. Here we present a case of NGT insertion in a patient with underlying coagulopathy, which led to aspiration of nasal bleeding, causing respiratory distress. This report aims to highlight a potentially severe complication of NGT tube insertion and to provide the physician with insight into preventing such events.

## Case presentation

An 81-year-old male with a history of mild cognitive deficits, chronic kidney disease, hypertension, heart failure with preserved ejection fraction, and type II diabetes mellitus presented to the emergency department with generalized weakness and vomiting. The patient initially had changes in his vital signs, including persistent bradycardia, and therefore was admitted to the Cardiac Care Unit (CCU) to manage presumed symptomatic junctional bradycardia and hyperkalemia. He was treated with a dopamine infusion and temporizing measures for his hyperkalemia, which was thought to be secondary to acute kidney injury superimposed on his chronic kidney disease. On hospital day 3, his CCU course included one hemodialysis session for hyperkalemia and uremia with an initial potassium level of 6.3 mMol/L and a blood urea nitrogen (BUN) level of 62 Mg/dL, upon which his potassium level normalized, and the bradycardia improved. The patient was then downgraded from the CCU to the internal medicine service.

On hospital day 7, a dobhoff nasogastric tube insertion was attempted due to poor oral intake overnight. However, after three unsuccessful attempts, the patient developed diffuse epistaxis and hematemesis. The nursing staff made no further attempts at NGT insertion at that point. After 12 hours, the patient’s mental status became more altered and less responsive to physical and verbal stimuli. A rapid response was called, and the patient was found to have a significant hemoglobin level drop to 6.0 g/dL, down from the previous value of 7.3 g/dL. A coagulation panel was performed, demonstrating coagulopathy with a prothrombin time of 12.5, an activated partial prothrombin time of 30, and an international normalized ratio (INR) of 1.20. In addition to this, it was found that the patient’s other cell lines were also less than the lower limit of normal consist with pancytopenia. The patient became hypotensive and received one packed red blood cell and 1 L normal saline bolus. Shortly after, the patient became unresponsive, with persistent tachypnea and hypotension. At this point, the medical intensive care unit was consulted for evaluation. Upon evaluation, the patient’s arterial blood gas analysis showed pH: 7.25 PaCO2: 50.5 PaO2: 75.5 HCO3: 23.5 with a fraction of inspired oxygen (FiO2) 100% with a non-rebreather mask (NRM). The patient was urgently transferred to the medical intensive care unit, and the decision was made to intubate the patient for airway protection.

**Figure 1:**
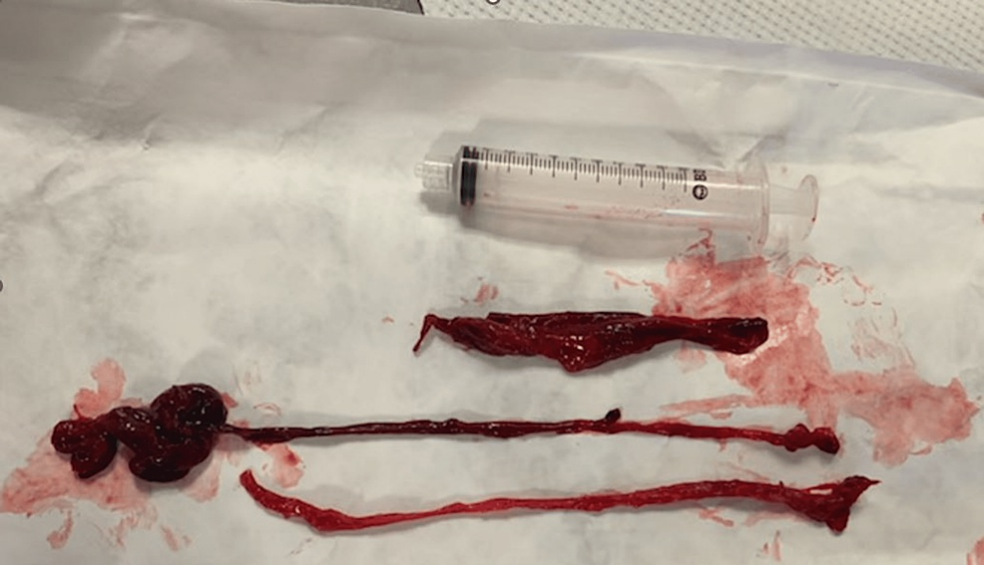
Three large blood clots removed during difficult intubation

The patient was pre-oxygenated with a bag-valve mask and induced with Etomidate and Rocuronium. Although we did not expect difficult intubation, the team routinely prepared a MAC2 Video-assisted device and Bougie at the bedside. Initial intubation was attempted using MAC 4 blade; however, the operator could not see the airway with obstructing object, so without any attempt, the tool was changed to a MAC2 video-assisted device; the device monitor showed a large mass entirely blocked the oropharynx, and the operator could not see the vocal cords. Three large blood clots were removed via aggressive suctioning and manual removal (Figure 1). Immediately after the clot removal, the epiglottis and vocal cords were visualized; however, they were considerably swollen, so cricothyroid pressure was applied, and a 7.5 endotracheal tube was advanced via Bougie assistance. Intubation was successful, with confirmation via an end-tidal CO2 monitor, bronchoscope, and chest X-ray. Post-intubation bronchoscopy confirmed correct endotracheal tube placement, absence of active hemorrhage and it was additionally used to clear the airway of mucus. The patient’s hemoglobin level improved after the administration of blood products. Due to the patient’s pancytopenia and mild coagulopathic state, investigation was initiated for this to include a comprehensive work-up which was negative. Otorhinolaryngology was consulted for the nasopharyngeal bleed; however, after ICU day 1, there was no evidence of active bleeding from his nasopharynx. After administration of blood products, the hemoglobin level normalized, and the subsequent hospital course did include persistent thrombocytopenia; however, it never lowered to levels that would need an additional transfusion.

On ICU day 7, the patient also had a resolution of his respiratory failure and was extubated on ICU day 12. The patient was then downgraded from the ICU to an intermediate level of care, where he had a relatively uncomplicated course and was subsequently discharged from the hospital.

## Discussion

Nose bleeding from nasogastric tube placement occurs when the mucosa is eroded and nasopharyngeal vessels become exposed and broken. This bleeding from traumatic NGT placement usually originates from an arterial source such as the Kiesselbach plexus or sphenopalatine arteries from mucosal erosion [6]. Usually, this bleeding can be controlled by supportive measures and does not cause any other downstream complications in the healthy patient. However, abnormal coagulation may increase the risk of nose bleeds from minimal pharyngeal trauma. Therefore NGT placement should be placed with caution by experienced medical staff and forceful or traumatic insertion should be avoided. Prior case studies have demonstrated this phenomenon, leading to difficult-to-control nasal bleeding [6]. One observational study reported that approximately 80% of epistaxis in coagulopathic patients was due to NGT insertion [7]. Notably, the source of the patient’s pancytopenia was evaluated with a full work-up; however, all laboratory analysis tested was negative, which pointed to a critical illness-induced pancytopenia seen often in this patient population [8]. The coagulopathy was also evaluated with appropriate work-up however no identifiable cause was established and concluded to be secondary to consumption from the nasopharyngeal bleed.

In patients with nasopharyngeal epistaxis, multiple treatments should be considered, including nasal packing, application of topical vasoconstrictive medications, and possible embolization of arteries that supply the region [9]. Currently, there are no established guidelines regarding complicated airway management in the setting of nasopharyngeal epistaxis-induced airway obstruction; recommendations do, however, suggest removing the obstruction and proceeding with securing the airway [10].

In cases like the one presented here, it is reasonable to predict that complications such as aspiration of blood content from a coagulopathic patient can lead to respiratory collapse and subsequent difficult intubation. In normal healthy adults, aspiration of blood can usually be mitigated by a cough reflex and, therefore, rarely obstructs airways. However, the patient presented in this case had an underlying mild cognitive deficit manifesting as dementia which can raise the risk of hospital/ICU-acquired delirium. This can diminish a patient’s cough reflex and other intrinsic airway-clearing mechanisms, thus increasing complications such as aspiration [9]. It is worth mentioning that in patients with underlying dementia/cognitive deficits, aspiration risks are much higher than regular healthy counterparts; however, even more importantly, it is possible that the patient was unaware of his nosebleed, and thus time to recognition of the complication was delayed. This underscores the importance of consideration of this complication when attempting to place the endotracheal tube.

It was appropriate in this case that the initial intubation attempt included a video-assisted device compared to direct laryngoscopy, as this could have led to additional time spent attempting to remove the blood clot obscuring the airway. Suction was utilized to remove the blood clot, followed by a second attempt with concomitant cricothyroid pressure applied due to visualizing the epiglottis and vocal cord edema. As presented in this case, a difficult airway requires the physician to anticipate and prepare for this, even in the emergent setting. Such situations require having all equipment prepared and ready in the room before the intubation attempt with secondary and tertiary emergent airway management plans set in place [10]. In this case, the intubation operator and medical staff were surprised to find difficult intubation; however, guidelines suggest always being prepared for such an event. Such practices include having the available equipment for difficult airway management in the room at the time of intubation, proper patient positioning, ensuring that a skilled individual is present or immediately available to assist with airway management, and having equipment for a surgical airway present at the bedside as a last resort effort [10]. In the setting of an anticipated difficult airway, the guidelines suggest implementing a video-assisted laryngoscope and airway maneuvers such as cricothyroid pressure application on the first attempt, which was performed in this case. Every physician should know these recommendations and be familiar with the adult patient’s ASA difficult airway algorithm [10].

## Conclusions

Although NGT placement is considered a reasonably safe procedure, certain conditions such as coagulopathy, a recent history of trauma to the upper airway, in-hospital delirium/baseline cognitive deficits, or a recent tracheostomy put patients at a relatively higher risk of complications. Given the cognitive deficit and delirium experienced by the patient presented in the case, it is possible that time to recognition of the mucosal bleed caused by forceful NG tube placement was delayed and resulted in a difficult airway.

## References

[1] O'Connell F, Ong J, Donelan C, Pourmand A (2021). Emergency department approach to gastric tube complications and review of the literature. Am J Emerg Med.

[2] Paul V, Kupfer Y, Tessler S (2013). Severe epistaxis after nasogastric tube insertion requiring arterial embolisation. BMJ Case Rep.

[3] Weinberg L, Skewes D (2006). Pneumothorax from intrapleural placement of a nasogastric tube. Anaesth Intensive Care.

[4] Sanaie S, Mahmoodpoor A, Najafi M (2017). Nasogastric tube insertion in anaesthetized patients: a comprehensive review. Anaesthesiol Intensive Ther.

[5] Shafik CG, Buck ML, de Faria Freitas AJ, Dixon BJ, Dhillon R (2021). Iatrogenic traumatic pontine injury from nasogastric tube insertion in a patient with an occult clival chordoma. ANZ J Surg.

[6] Camus M, Jensen DM, Matthews JD (2014). Epistaxis in end stage liver disease masquerading as severe upper gastrointestinal hemorrhage. World J Gastroenterol.

[7] Kim YH, Lim BG, Yoon YS, Hong SY, Lee YS, Kim WY, Don JH (2016). Nasogastric tube induced refractory epistaxis during liver transplantation. Adv Biosci Clin Med.

[8] Woźnica EA, Inglot M, Woźnica RK, Łysenko L (2018). Liver dysfunction in sepsis. Adv Clin Exp Med.

[9] Viehweg TL, Roberson JB, Hudson JW (2006). Epistaxis: diagnosis and treatment. J Oral Maxillofac Surg.

[10] Apfelbaum JL, Hagberg CA, Connis RT (2022). 2022 American Society of Anesthesiologists practice guidelines for management of the difficult airway. Anesthesiology.

